# In Vitro Evaluation of Antioxidant Potential of the Invasive Seagrass *Halophila stipulacea*

**DOI:** 10.3390/md19010037

**Published:** 2021-01-16

**Authors:** Clementina Sansone, Christian Galasso, Marco Lo Martire, Tomás Vega Fernández, Luigi Musco, Antonio Dell’Anno, Antonino Bruno, Douglas M. Noonan, Adriana Albini, Christophe Brunet

**Affiliations:** 1 Stazione Zoologica Anton Dohrn, Istituto Nazionale di Biologia, Ecologia e Biotecnologie Marine, Villa Comunale, 80121 Napoli, Italy; christian.galasso@szn.it (C.G.); tomas.vegafernandez@szn.it (T.V.F.); luigi.musco@szn.it (L.M.); christophe.brunet@szn.it (C.B.); 2 Department of Life and Environmental Sciences, Università Politecnica delle Marche, Via Brecce Bianche, 60131 Ancona, Italy; m.lomartire@univpm.it (M.L.M.); a.dellanno@staff.univpm.it (A.D.); 3 Unit of Immunology, IRCCS MultiMedica, 20138 Milan, Italy; antonino.bruno@multimedica.it; 4 Department of Biotechnology and Life Sciences, University of Insubria, 211000 Varese, Italy; douglas.noonan@uninsubria.it; 5 Laboratory of Vascular Biology and Angiogenesis, IRCCS MultiMedica, 20138 Milan, Italy; adriana.albini@multimedica.it

**Keywords:** oxidative stress, seagrass, carotenoids, scavenging effect, repair activity

## Abstract

Marine organisms with fast growth rates and great biological adaptive capacity might have biotechnological interests, since ecological competitiveness might rely on enhanced physiological or biochemical processes’ capability promoting protection, defense, or repair intracellular damages. The invasive seagrass *Halophila stipulacea*, a non-indigenous species widespread in the Mediterranean Sea, belongs to this category. This is the premise to investigate the biotechnological interest of this species. In this study, we investigated the antioxidant activity in vitro, both in scavenging reactive oxygen species and in repairing damages from oxidative stress on the fibroblast human cell line WI-38. Together with the biochemical analysis, the antioxidant activity was characterized by the study of the expression of oxidative stress gene in WI-38 cells in presence or absence of the *H. stipulacea* extract. Concomitantly, the pigment pool of the extracts, as well as their macromolecular composition was characterized. This study was done separately on mature and young leaves. Results indicated that mature leaves exerted a great activity in scavenging reactive oxygen species and repairing damages from oxidative stress in the WI-38 cell line. This activity was paralleled to an enhanced carotenoids content in the mature leaf extracts and a higher carbohydrate contribution to organic matter. Our results suggest a potential of the old leaves of *H. stipulacea* as oxidative stress damage protecting or repair agents in fibroblast cell lines. This study paves the way to transmute the invasive *H. stipulacea* environmental threat in goods for human health.

## 1. Introduction

Antioxidants have a key role in counteracting free radicals, preventing or limiting damages in living organisms [1]. Exogeneous antioxidants are requested to enhance the protection or repairing processes, with a special regard on natural antioxidative compounds [2]. Photosynthetic organisms represent the most important source of natural products, providing health benefits [3], with plant-derived compounds used in clinical practice as anticancer, anti-inflammatory, antibiotic, or other medicinal drugs [3,4]. Despite the high genetic and phenotypic diversity of algae and seagrasses, and their increasing biotechnological interests [5,6,7,8], marine photosynthetic organisms are less exploited than the land ones. In the marine scenario, the bioactive profile of seagrasses is less investigated than algae [9], although diverse studies addressed the potential of seagrasses, such as *Halophila* spp. [10,11].

However, it is not conceivable nor sustainable using seagrasses harvested from their habitats as a biotechnological resource, because of the damageable consequences on local ecosystem functioning.

Conversely, in case of invasive species this paradigm becomes not even more true. This is the case of the invasive seagrass *Halophila stipulacea* in the Mediterranean sea [12,13], which belongs to the “100 Worst Invasive Alien Species in the Mediterranean” [14]. In the Mediterranean sea, *H. stipulacea* (Forsskål and Niebuhr) Ascherson (order Alismatales, family *Hydrocharitaceae*) is replacing the seagrass *Cymodocea nodosa* [15], concurrently with the ongoing “tropicalization” of the Mediterranean basin [16].

The potential bioactivity and biological properties of extracts from *Halophila stipulacea* was previously investigated [10,17]. This species—i.e., extracts from leaves or stems—exerted a reduction of lipid content in fatty acid-overloaded liver cells (steatosis) and displayed antifouling activity [17]. Moreover, ethanolic leaf extracts showed antioxidant and antibacterial activities [18].

In relation with these premises, ecologically negative properties relied on *H. stipulacea* invasiveness while being biotechnologically attracting; the aim of this study was to seek for human health bioactivity interests of *H. stipulacea*. The general hypothesis behind this study was to pave the way to transform this ecosystem-harmful species in benefit for humans. In this context, we investigated the interest of *H. stipulacea* as a source of antioxidant for scavenging and/or repairing oxidative stress carrying out in vitro analysis on human cell line. *H. stipulacea* was sampled in the Sicily coast (Mediterranean sea) in summer, i.e., when environmental conditions (high light, UVs, low nutrient concentration, high temperature) promote the activation of intracellular acclimation strategies coping with stressful external forcing [19,20]. The bioactivity of the seagrass was assessed on WI-38 lung fibroblast cell line, which is frequently used as an in vitro model for studying toxicity, oxidative stress, and aging-related disorders [21,22,23,24]. Bioactivity analysis was integrated with the pigments content and the macromolecular composition of the extracts. This investigation was done comparing the results obtained separately on old and young leaves of *H. stipulacea*, to address the role of leaf aging on the overall bioactive properties [25]. Indeed, old leaves displayed a significantly higher antioxidant capacity and ability to scavenge and repair epithelial cells from oxidative stress. This result is concomitant with the much higher content of carotenoids, mainly xanthophylls with photoprotective and/or antioxidant role. The interesting bioactivity pattern displayed by *H. stipulacea* makes this species potentially exploitable, with the objective to transform the actual *H. stipulacea*-induced environmental threat into a biotechnological output. However, the exploitation of *H. stipulacea* as a source of wellness, however, still requires technological development and strategy for the control and removal of this invasive species [26,27].

## 2. Results

### 2.1. 2,2,1-Diphenyl-1-Picrylhydrazyl (DPPH) Radical Scavenging Activity

**Figure 1:**
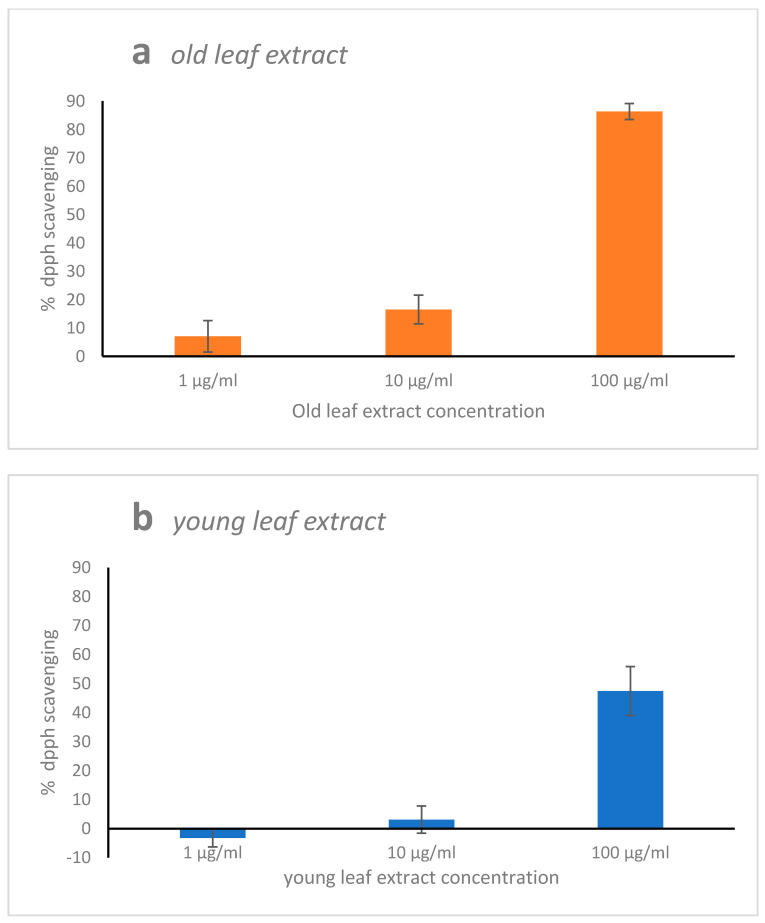
DPPH scavenging activity of the old (**a**) and young (**b**) leaf extracts of *H. stipulacea* at three concentrations (1, 10, and 100 μg·mL^−1^). Values represent the % of inhibition of free radical DPPH. Data are shown as mean ± S.D. All means were significantly different from each other (*p* < 0.01).

Ethanol/water mixture (3/1; *v*/*v*) *H. stipulacea* extracts from old leaf reached 85% of inhibition of free radical DPPH, displaying a significant (*p* < 0.001) higher antioxidant capacity than extracts from young leaf at any of the three tested extract concentrations (Figure 1a). The free radical scavenging of the young extracts was generally very low, with a maximum of 45% at the higher extract concentration (Figure 1b).

### 2.2. Cytotoxicity, Scavenging, and Repair Activities of the Leaf Extracts

**Figure 2:**
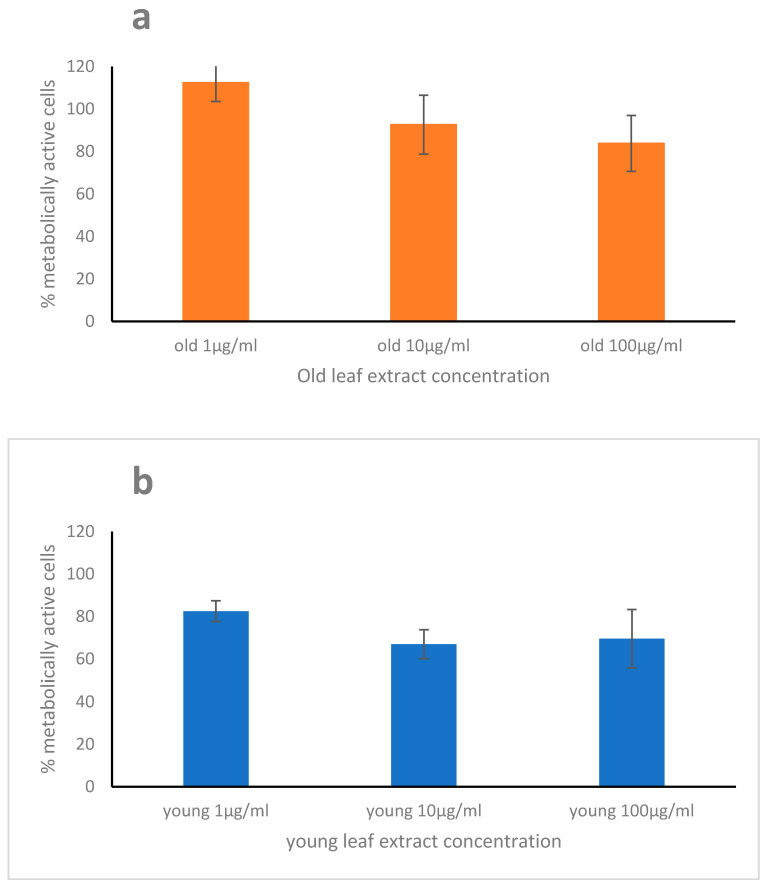
Cytotoxicity of old (**a**) and young (**b**) leaf extract of *Halophila stipulacea* on the WI38 cell line. The WI-38 cells were treated with three concentrations (1, 10, and 100 μg·mL^−1^) of the extracts for 48 h. Data are shown as mean ± S.D. The means for all the three concentrations were significantly different between old and young leaf extracts (*p* < 0.001).

While the old leaf extract of *H. stipulacea* induced a low cytotoxicity on WI-38 cells (Figure 2a) at the highest leaf extracts concentration, the young leaf extract displayed a slight cytotoxicity on WI-38 cells (Figure 2b) already at the lowest concentration, with a metabolically active cell percent decreasing at 83% (at 1 μg·mL^−1^) and at around 68% (at 10 and 100 μg·mL^−1^).

**Figure 3:**
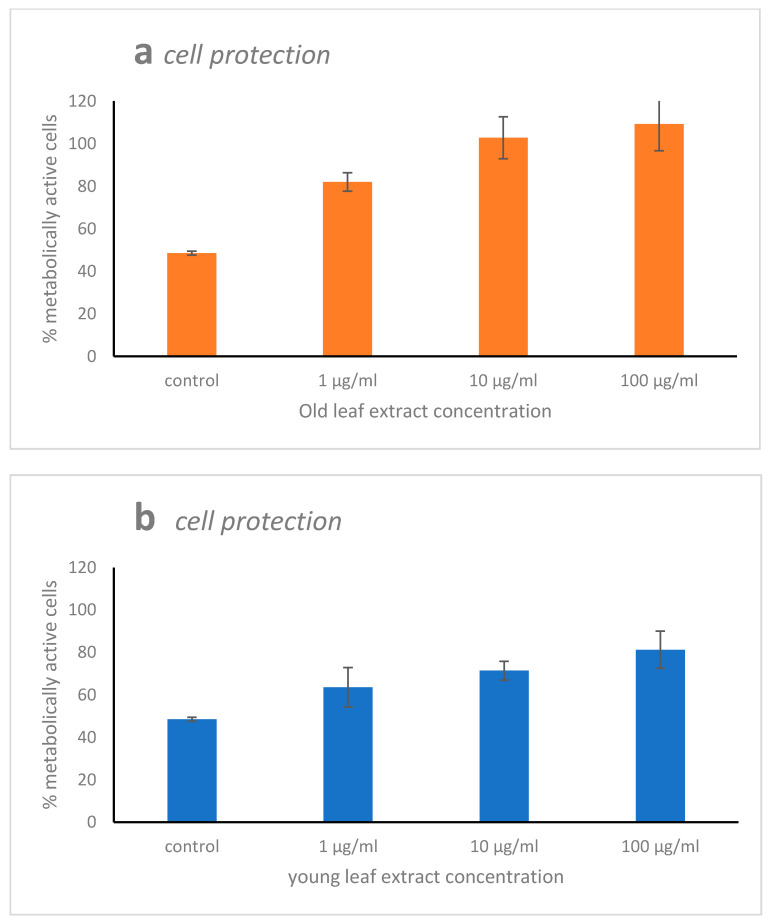
Cell protection activity of old (**a**) and young (**b**) leaf extract of *Halophila stipulacea* on the WI38 cell line. The WI-38 cells were pre-treated for two hours with three concentrations of the extracts (1, 10, and 100 μg·mL^−1^) and then injured with 10 mM of H_2_O_2_ for 48 h. Data are shown as mean ± S.D. The means for all the three concentrations were significantly different between old and young leaf extracts (*p* < 0.001).

WI-38 cells injured by 10 mM of H_2_O_2_ for 48 h strongly lowered the metabolically active cell percent (48%). The pre-treatment of the human cell line with *H. stipulacea* extract helped cells to protect themselves against H_2_O_2_ injury. Indeed, the pre-treatment of WI-38 cells with old leaf extract enhanced the metabolically active cell percent to 82%, 102%, and 109% at the three extract concentrations (1, 10, and 100 μg·mL^−1^), respectively (Figure 3a). Adding young leaf extract to WI-38 cells as pre-treatment, the metabolically active cell percent enhanced in a lower way than with the old leaf extracts, “only” reaching 63%, 71%, and 81% for the three extract concentrations (1, 10, and 100 μg·mL^−1^), respectively (Figure 3b).

**Figure 4:**
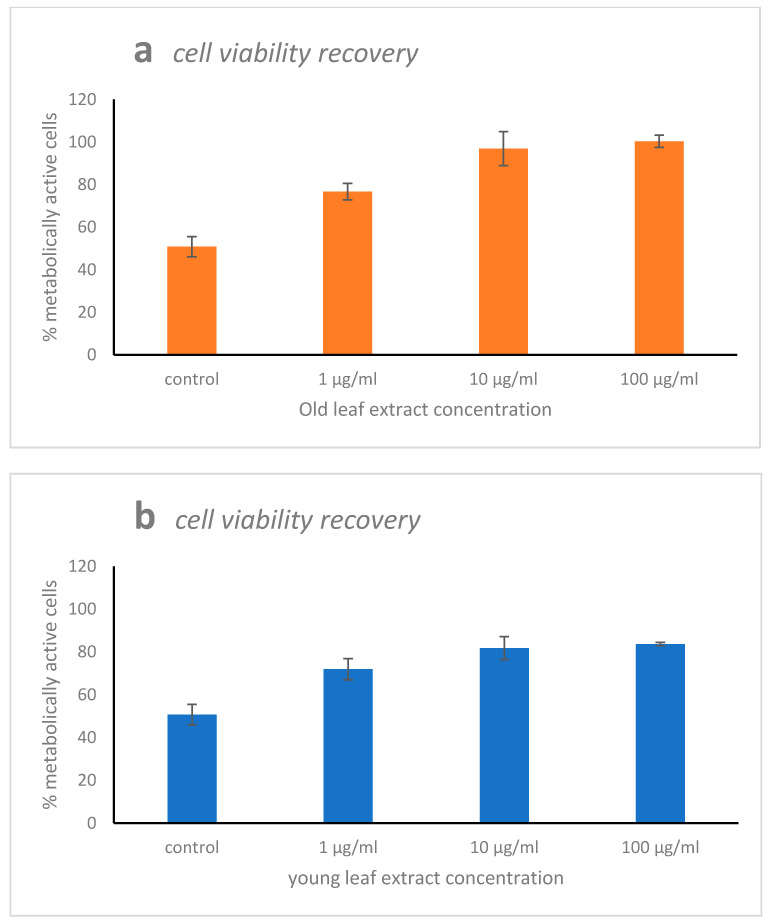
Cell viability recovery of old (**a**) and young (**b**) leaf extract of *Halophila stipulacea* on the WI38 cell line. The WI-38 cells were injured with 10 mM of H_2_O_2_ for 2 h and then recovered with three concentrations of the extracts (1, 10, and 100 μg·mL^−1^) for 48 h. Data are shown as mean ± S.D. The means for the two greatest leaf extract concentrations (10 and 100 μg·mL^−1^) were significantly different between old and young leaves (*p* < 0.001). No significant difference (*p* > 0.05) between old and young leaf extracts at the lowest concentration (1 μg·mL^−1^).

The positive control of the “cell viability recovery” experiment, represented by WI-38 cells injured with 10 mM of H_2_O_2_ for 1 h displayed around 50% of metabolically active cells (Figure 4a,b). Recovery treatment applying concentrations of 1, 10, or 100 μg·mL^−1^ of the old leaf extract enhanced the percentage of metabolically active cells percent to 67, 97, and 100%, respectively (Figure 4a). Adding young leaf extract as potential recovery agent, the metabolically active cell percent increased to 71%, 81%, and 83%, respectively (Figure 4b).

### 2.3. Oxidative Stress Gene Expression in WI-38 Cells Treated with Old Leaf Extracts

In order to assess the intracellular response at gene expression level, we performed a PCR Array experiment by analyzing the 84 genes involved in oxidative stress defense. Here we showed the fold regulation of the key genes involved in antioxidant pathways, all genes analyzed are listed in Supplementary Materials (Tables S1 and S2). WI-38 cells treated with 10 mM of H_2_O_2_ exhibited an upregulation of the genes epoxide hydrolase 2 (EPHX2), eosinophil peroxidase (EPX), mannose-binding lectin 2 (MBL2), myeloperoxidase (MPO), and serine peptidase inhibitor-Kazal type 1 (SPINK1), (3.0, 2.1, 7.0, 8.8, and 33.7 in fold regulation, respectively) and the downregulation of Cytochrome b-245 (CYBB) (−38 in fold regulation) (Tables S1 and S2). On the contrary, old leaf extract induced an up-regulation of the CYBB gene (3.9 in fold regulation) and a down-regulation of EPHX2, EPX, MBL2, MPO, and SPINK1 genes (−19, −2, −11.6, −6.9, and −10 in fold regulation, respectively) indicating that the oxidative stress response is not activated when cells were treated with *H. stipulacea* (Tables S1 and S2).

**Figure 5:**
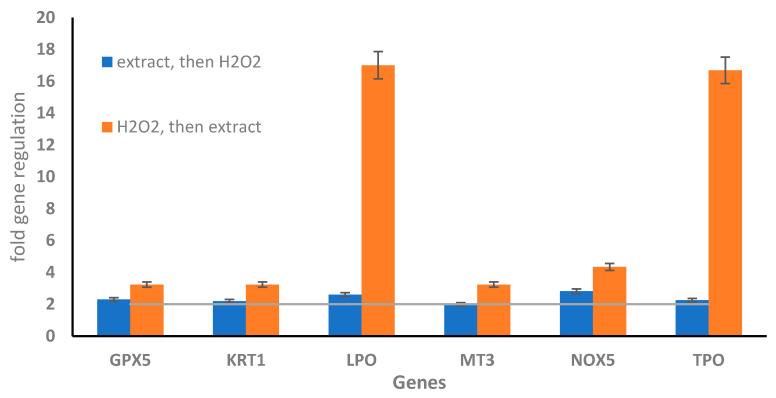
Effect of old leaf extract (10 μg mL^−1^) on the regulation of oxidative stress gene expression in H_2_O_2_-treated human normal lung fibroblasts (WI-38). WI-38 cells treated with the old leaf extract and then injured with H_2_O_2_ (cell protection activity; blue bars) and WI-38 treated with H_2_O_2_ and then with the old leaf extract (cell recovery treatment; orange bars). Expression values greater or lower than a two-fold difference with respect to the controls were considered significant (horizontal line indicates the ± two-fold differences with respect to controls). Data are shown as mean ± S.D.

Interestingly when the WI-38 cells were pre-treated with old leaf extract before injury with 10 mM of H_2_O_2_, an upregulation of all genes involved in the antioxidant cell response analyzed, such as glutathione peroxidase 5 (GPX5, 2.3 fold regulation), keratin 1 (KRT1, 2.2 fold regulation), lactoperoxidase (LPO, 2.6 fold regulation), metallothionein 3 (MT3 2.0 fold regulation), NADPH oxidase 5 (NOX5 2.8 fold regulation), and thyroid peroxidase (TPO 2.3 fold regulation) (Figure 5).

Cell recovery treatment with old leaf extract after the injury with H_2_O_2_ induced a significant increase in fold regulation of the genes glutathione peroxidase 5 (GPX5, 3.2 fold regulation), keratin 1 (KRT1, 3.4 fold regulation), lactoperoxidase (LPO, 17 fold regulation), metallothionein 3 (MT3 3.3 fold regulation), NADPH oxidase 5 (NOX5 4.3 fold regulation), and thyroid peroxidase (TPO 16.7-fold regulation). These results probably indicated that *H. stipulacea* old leaf extract could be more therapeutic than preventive against peroxidative damage of the GSR gene (Figure 5 and Tables S1 and S2).

### 2.4. Pigments

**Figure 6:**
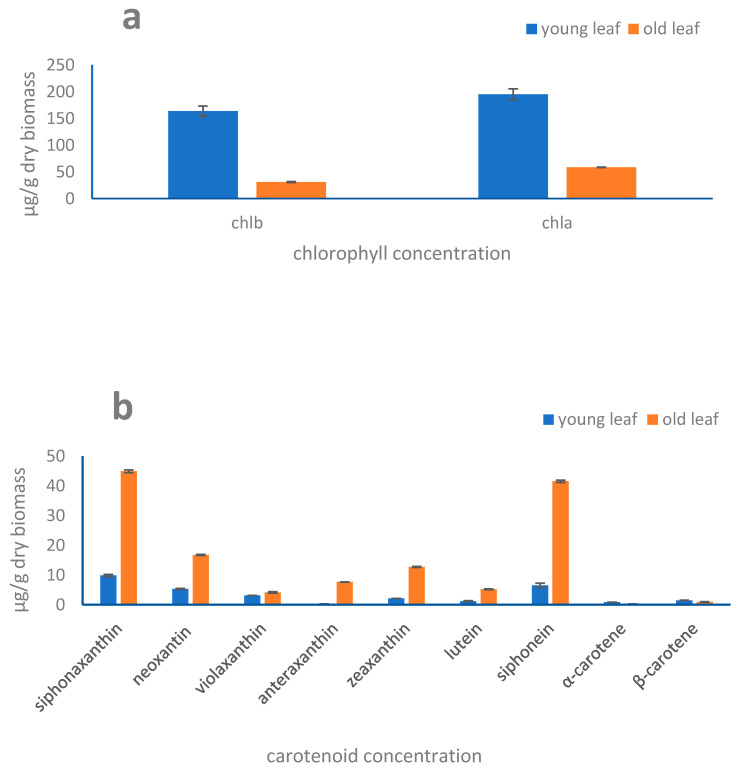
Pigment analysis in young and old leaves of *H. stipulacea.* (**a**) Concentrations (μg·g^−1^ dry biomass) of chlorophylls *a* and *b*; (**b**) concentrations (μg·g^−1^ dry biomass) of violaxanthin, antheraxanthin, zeaxanthin, neoxantin, lutein, siphonein, siphonaxanthin, α- and β-carotenes. Values are reported as means of triplicates with standard deviation. All pigment concentrations were significantly different between old and young leaf extracts (*p* < 0.001), except violaxanthin, α- and β-carotenes (*p* > 0.05).

Pigment composition was similar in old and young leaves: the main pigments revealed in *H. stipulacea* belong to the two photoprotective cycles, violaxanthin, antheraxanthin, and zeaxanthin (xanthophyll cycle), and the siphonein-siphonaxanthin cycle, together with other carotenoids neoxanthin, lutein, α- and β-carotenes, and the chlorophylls *a* and *b* (chl.*a* and chl.*b*). Their concentration significantly varied with leaf aging (Figure 6a,b). The concentrations of the xanthophyll-cycling pigments, antheraxanthin and zeaxanthin, as well as siphonein and siphonaxanthin concentration pigments were significantly higher in old leaves than in the young ones (*p* < 0.001). Conversely, chl.*a* concentration was significantly higher in young than in old leaves (*p* < 0.01) while the α- and β-carotenes concentration was similar in young and old leaves (Figure 6a,b). Except for chl.*b* and the α- and β-carotenes, all the accessory pigments vs. chl.*a* ratios were significantly higher in old leaves compared to the young ones (*p* < 0.001; data not shown), with the photoprotective antheraxanthin and zeaxanthin displaying the greatest increase with leaf aging.

### 2.5. Macromolecular Composition

Protein, carbohydrate, and lipid concentrations in young leaves were significantly higher than in old ones (*p* < 0.05; Table 1). Protein concentrations decreased from 21.3 ± 0.5 to 8.2 ± 0.6, carbohydrates from 19 ± 0.6 to 10.9 ± 1.1, and lipids from 5.8 ± 0.1 to 1.6 ± 0.3 mg·g^−1^ in young and old leaves, respectively (Table 1).

Interestingly, the relative contribution of these three macromolecular families varied in old vs. young leaves. Aging enhanced the carbohydrates contribution which represented the dominant component (accounting for 52.6% of the total organic matter pool) followed by proteins and lipids (39.6% and 7.8%, respectively). Conversely, in young leaves, proteins represented the main macromolecular components contributing to 46.2 % of the total organic matter pool, followed by carbohydrates (41.2%) and lipids (12.6%).

## 3. Discussion

The seagrass *H. stipulacea* was selected as target in our study because of its capacity to thrive in a wide range of environmental conditions and to out-compete native species in the Mediterranean Sea, where it is invasive [28,29,30,31]. The opportunism and/or competitiveness of organisms such as *H. stipulacea* is based on their great physiological and biochemical plasticity for maintaining high growth rate also under new (favorable) environmental conditions. This implies relevant biochemical energy conveyed toward growth as well as efficient processes to protect cells and repair damages in case of non-optimal environmental conditions. This is likely to occur even more during summer under environmental conditions (e.g., high light, UV radiations, low nutrient concentration, high temperature) that promote the onset of intracellular regulative strategies to cope with stressful external forcing [19,20]. Those characteristics enhance the role of organisms as putative targets for biotechnological prospecting to seek for bioactive natural compounds/extracts with human health benefits, such as antioxidants. Indeed, the invasive seaweed *Sargassum muticum* displayed higher DPPH and antioxidant activity than the native seaweed *Bifurcaria bifurcata* in the coastal area of Portugal [32]. Also, results obtained in this study highlight the high potential of the seagrass *H. stipulacea* as a source of bioactive extracts able to counteract and repair fibroblast cells from induced-oxidative stress. DPPH free radical scavenging activity reported in our study for old leaf extracts of *H. stipulacea* is much higher than results reported from other seagrasses [18,32,33,34,35] and likely similar than those reported for *Cymodocea rotundata* [35].

While young leaf extracts are not attractive, old leaf ones are, conversely, biotechnologically appealing. Organismal ageing induces a senescence process that accentuates the modulation of physiology, biochemistry, and functioning of biological machinery [36]. For instance, the higher contribution of carbohydrates into the organic matter content, detected in our study, is a common feature in plants during ageing, potentially promoting or triggering senescence [37,38,39,40,41]. The decrease in the chlorophyll concentration in old leaves, as already observed [42] might induce a lowering of light energy transfer in the cells, that in turn would explain the lower organic matter content in aged leaves compared to the young leaves. Senescence is regulated by many processes [36] and by ROS, which acts as signaling for production of secondary metabolites involved in several defense and protective mechanisms [43,44]. Even though concentration of carotenoids might remain stable during ageing/senescence in some plants [45], growth stage of a photosynthetic organism notably affects the bioactive compounds content and antioxidant activity often increasing in aged biological material [20,46,47,48,49,50]. In *H. stipulacea*, the enhancement of antioxidant ability with leaf aging is paralleled to an almost all carotenoids increase and with the decrease in chl.*a* content. The significant increase in carotenoids with age might—at least in part—explain the enhanced antioxidant power of the extracts. Indeed, carotenoids, such as violaxanthin, neoxanthin, antheraxanthin, zeaxanthin, lutein are epidemiologically correlated with a lower risk for several diseases and to have beneficial effects on eye health [51,52,53]. Additionally, *H. stipulacea* contains the keto-carotenoids siphonein and siphonaxanthin, exclusively present in aquatic photosynthetic organisms, as already found in green macroalgae such as *Codium fragile*, *Caulerpa lentillifera*, and *Umbraulva japonica* [54]. These pigments fill the role of maximizing the absorption of the green and blue-green wavelengths [47]. Moreover, they are known to act as defense agents against light stress [47], being active in photoprotection processes [20,48]. The two carotenoids siphonein and siphonaxanthin have been reported as bioactive compounds [55]. Siphonoxathin is also known to induce apoptosis in leukemia cells [55], with additional anti-angiogenic or anti-diabetic role [56,57].

The bioactivity of the old leaf extracts on human fibroblast cells is greatly promising with a recovery of cell viability at around 100% coping (protecting or recovering) with the stress due to H_2_O_2_ that caused a reduction in cell viability to 48%. This activity induced by the old leaf extracts involves a molecular response turned toward the cell protection and detoxification from peroxidative cell damages. The study of the oxidative stress gene expression patterns between cells treated with 10 mM H_2_O_2_ and those pre-treated or recovered with 10 μg·mL^−1^ extract reveals an interaction with the ROS metabolism, with an up-regulation of the oxidative stress responsive genes GPX5, KRT1, LPO, MT3, NOX5, and TPO during the recovery treatment. All these genes, together with CCL5 and MBL2 are known to activate intracellular detoxification pathways in response to oxidative stress, with the involvement of the peroxisome’s machinery [58]. In particular, antioxidant gene such as GPX5 displayed an opposite regulative expression behavior when the extract was added before and after H_2_O_2_ (Table S2). On the contrary, the up-regulation of EPHX2 with H_2_O_2_ is due to the fact that this gene codes for bifunctional enzyme in response to oxidative stress [59]. The recovery treatment with extract induces a lowering of EPHX2 gene expression until to be down-regulated at 10 μg·mL^−1^ (Table S2). This is probably due to the ability of the extract to scavenge the effect induced by H_2_O_2_, thereby reducing EPHX2 gene expression levels. Also, NOX5 involved in ROS metabolism is up-regulated by the extract as already reported in relation with the effects of vasoactive agents, growth factors, and pro-inflammatory cytokines protecting cells against oxidative stress [60]. This is linked to the function of this gene which encodes for a protein localized in the perinuclear and endoplasmic reticulum regions of cells and traffics to the cell membrane upon activation. Another relevant result—and effects of the old leaf extract—regards the up-regulation of LPO and TPO genes, both involved in the resistance against oxidative stress and preventing apoptotic cell death when they are expressed at high levels [61].

This study highlights the pro-active effect of *H. stipulacea* leaf extracts against damaged human cells or protecting them, owing to an induced-gene activation cascading effect. Advanced chemical investigations might further help in relating the reported bioactivity to the compounds’ diversity collected in the extract, even though the role of carotenoids—in term of quantity and diversity, the latter enhancing the bioactive ability of the extract—is well-known.

## 4. Materials and Methods

### 4.1. Halophila Stipulacea Sampling and Leaf Collection

Rhizomes of *H. stipulacea* were collected in July 2016 in the harbor of Castellammare del Golfo in the Mediterranean Sea (38°01′45.2′′ N, 12°52′51.8′′ E) at 0.5 m depth. The seagrass formed a dense patch of c.a. 1.5 m diameter on muddy sand bottom, exposed to direct sunlight, in an area characterized by urban run-off. Three seagrasses were sampled, and after collection, the fronds were gently rinsed in seawater to remove the sediment particles and associated epibionts and transported in seawater tanks to the laboratory for the subsequent analyses.

Young and mature old leaves were separated, the former being closer to the apex than the old leaves (more toward the bottom). From each seagrass, three young and mature leaves were collected and pooled together.

### 4.2. Preparation of Ethanol/Water Extracts from Halophila Stipulacea Leaves

Extracts for pigment analysis and biological assays were obtained from freeze-dried young and old leaves of *H. stipulacea*, according to Sansone et al. [62] at room temperature, under dark and N_2_ atmosphere to avoid oxidation during procedure. The fresh and dry weight of the leaf samples was measured, with a ratio between dry and fresh ≈0.135 (±0.030).

One gram of dried leaves was placed in 1 mL of ethanol/water mixture (3/1; *v*/*v*) for 30 min in dark condition and mechanically disrupted with a pestle. The mixture was separated by centrifugation at 4500× *g* for 15 min, at 4 °C, and the upper layer was transferred to a clean tube. Pellets were resuspended in 1 mL of ethanol/water mixture to repeat the procedure, maximizing the extraction yield. Hydroalcoholic extracts were dried in a rotary vacuum evaporator (Buchi rotavapor R-114) and the dried extracts were stored at −20 °C.

### 4.3. Pigment HPLC Analysis

Pigment analysis was conducted by high performance liquid chromatography (HPLC), according to the method described in Smerilli et al. [63]. Prior to injection into the HPLC, 250 µL of an ion pairing agent (ammonium acetate 1 mol·L^−1^, final concentration of 0.33 mol·L^−1^) was added to 0.5 mL of the lyophilized extracts and incubated for 5 min in the dark at 4 °C. These samples were then injected in the 50 µL loop of the Hewlett Packard series 1100 HPLC (Hewlett Packard, Wilmington, NC, USA), equipped with a reversed-phase column (C8 Kinetex column; 50 mm × 4.6 mm; 2.6 µm particle size, Phenomenex^®^, Torrance, CA, USA). The temperature of the column was steadily maintained at 20 °C and the flow rate of the mobile phase was set up at 1.7 mL·min^–1^. The mobile phase was composed of two solvent mixtures: “A”—methanol/aqueous ammonium acetate (70/30, *v*/*v*); “B”—methanol. During the elution (12 min), a gradient between the solvents was programmed: 75% A (0 min), 50% A (1 min), 0% A (8 min) isocratic for 3 min. Chlorophylls and carotenoids were detected by diode-array spectroscopy (spectrum data collected in the range 350–750 nm) using a Hewlett Packard photodiode array detector, model DAD series 1100 and absorbance chromatogram was reported at 440 nm. Chlorophylls, and their degradation products were also detected by fluorescence using a Hewlett Packard standard FLD cell series 1100 with excitation and emission wavelengths set at 407 nm and 665 nm, respectively. Identification and quantification of pigments were carried out using pigment standards from the D.H.I. Water & Environment (Horsholm, Denmark).

### 4.4. Macromolecular Composition of the Extracts

The concentration of proteins, carbohydrates, and lipids was determined for the young and old leaves which were previously freeze dried. Total protein concentrations were determined according to Lowry et al. [64] and modified [65,66] to compensate for phenol interference and are expressed as bovine serum albumin (BSA) equivalents. Total carbohydrate concentrations, expressed in glucose equivalents, were obtained according to the Gerchacov and Hatcher [67] protocol based on the phenol and concentrated sulfuric acid reaction with saccharides. Total lipids were extracted in chloroform: methanol (1:1, vol:vol) [68], and the resulting fraction, after evaporation in a dry hot bath at 80 to 100 °C for 20 min, was quantified according to the sulfuric acid carbonization procedure [69]. Total lipids were expressed in tripalmitine equivalents. Protein, carbohydrate, and lipid concentrations were expressed as mg·g^−1^ of dry weight.

### 4.5. DPPH Free Radical Scavenging Activity

Three concentrations of the extracts (1, 10, and 100 µg/mL) were tested for the radical scavenger assay. These samples were mixed in a 96-well plate with a final concentration of DPPH of 0.1 mM in methanol, allowed to react for 30 min in the dark. The methanol solution was used as negative control. At the end of incubation, absorbance was measured at 517 nm, using a microplate reader. The scavenging assay was performed in triplicate. Results were presented as a percentage of DPPH reduction with respect to the methanol negative control.

### 4.6. Human WI-38 Cell Line Culture and Treatments

WI-38 is a diploid human cell line composed of fibroblasts derived from lung tissue of a 3-month-gestation aborted female fetus. This cell line was isolated by Leonard Hayflick in the 1960s and represents an easy cell model of normal human tissue [70]. WI-38 cell line was purchased from the American Type Culture Collection (ATCC^®^ CCL-75™) and grown in MEM (Eagle’s minimal essential medium) supplemented with 10% (*v*/*v*) fetal bovine serum (FBS), 100 units mL^−1^ penicillin, 100 units mL^−1^ streptomycin, 2mM of L-glutamine, and non-essential amino acids (NEAA, 2 mM) in a 5% CO_2_ atmosphere at 37 °C.

WI-38 cells (2 × 10^3^ cells well^−1^) were seeded in 96-well plates and kept overnight for attachment. The ethanol/water dried extracts, dissolved in dimethyl sulfoxide (DMSO, final concentration 1%), were used for cell treatments. The toxicity of vehicle (DMSO) was tested previously determining that the IC50 is around 7%, lower concentrations did not affect WI38 cell viability.

Seventy percent confluent cells were treated with young leaf- and old leaf- ethanol/water extracts (three replicates for each) at 1, 10, and 100 μg·mL^−1^ for 48 h, in order to assess cytotoxicity (see below) of the extracts and to run the experiments.

In one experiment, cells were pre-treated with the extracts at three concentrations (1, 10, and 100 μg·mL^−1^) for 2 h and then injured with 10 mM of H_2_O_2_ for 48 h. This experiment aimed to assess the potential scavenging activity of the extracts. The positive control was obtained injuring cells with only 10 mM of H_2_O_2_ for 48 h.

Another experiment was carried out as follow: the WI-38 cells were pre-injured for 2 h with 10 mM of H_2_O_2_ and then recovered with the extracts at the same three concentrations as before (1, 10, and 100 μg·mL^−1^) for 48 h. This experimental approach aimed to assess the potential repair activity of the extracts after cell oxidative damage. In this case, the positive control corresponded to pre-injuring cells for 2 h with 10 mM of H_2_O_2_ and then recovered with only culture medium.

### 4.7. Cell Viability Assays

Cytotoxicity, scavenging, and repair activity of the ethanol/water extracts were tested using the MTT (3-(4,5-Dimethylthiazol-2-yl)-2,5-Diphenyltetrazolium Bromide, Applichem, Darmstadt, Germany) on the WI-38 cell line [71]. After 48 h of treatment, WI-38 cells were incubated with 10 µL (5 mg·mL^−1^) of MTT and incubated for 3 h at 37 °C with 5% CO_2_. The resulting formazan crystals produced by viable cells were dissolved with 100 µL of isopropyl alcohol. The absorbance was recorded on a microplate reader at a wavelength of 570 nm. Effect of the extracts was evaluated as percent of metabolically active cells estimated as the ratio between absorbance of each sample and absorbance of control (untreated cells). Three different extracts for the two leaf categories (young vs. mature) were analyzed.

### 4.8. RNA Extraction and Real-Time qPCR

WI-38 cells (2 × 10^6^ cells), used for RNA extraction and analysis were seeded in Petri dishes (100 mm diameter). WI-38 cells were treated with old leaf extract (concentration at 10 μg·mL^−1^, displaying great repair and scavenging activities without any cytotoxic effect) and with 10 mM H_2_O_2_ as cellular injury. Experimental strategy was the following: WI-38 cells treated with the leaf extract (3 h); WI-38 cells treated with the leaf extract (2 h) and then injured with H_2_O_2_ (1 h); WI-38 cells injured with H_2_O_2_ (1 h) and recovered with the leaf extract (2 h). A negative control was setup with WI-38 cells without any treatment. Sampling for gene expression analysis was done after 3 h of treatment. Once the treatment was over, WI-38 cells were washed in the Petri dish by adding cold phosphate-buffered saline (PBS) and rocking gently. Cells were lysed in the Petri dish by adding 1 mL of Trisure Reagent (Bioline, Memphis, TN, USA) and RNA was isolated according to the manufacturer’s protocol. RNA concentration and purity were assessed using the nanophotomer NanodroP. The reverse transcription reaction was carried out with the RT^2^ first strand kit (Qiagen, Hilden, Germania), and real-time quantitative polymerase chain reaction (RT-qPCR) was performed using the RT^2^ Profiler PCR Arrays kit (Cat. No. PAHS-065Y, Qiagen). Plates were run on a ViiA7, Standard Fast PCR Cycling protocol with 10 µL reaction volumes. Cycling conditions were 1 cycle initiation at 95 °C for 10 min followed by amplification for 40 cycles at 95 °C for 15 s and 60 °C for 1 min. Amplification data were collected via ViiA 7 RUO Software (Applied Biosystems, Foster, CA, USA). The cycle threshold (Ct)-values were analyzed with PCR array data analysis online software (GeneGlobe Data Analysis Center, http://pcrdataanalysis.sabiosciences.com/pcr/arrayanalysis.php, Qiagen). Real time data were expressed as fold expression, describing the changes in gene expression between treated cells and untreated cells (control). The expression of 84 genes involved in oxidative stress was analyzed (Table S1). Control genes for real-time qPCR were actin-beta (ACTB), beta-2-microglobulin (B2M), hypoxanthine phosphoribosyl transferase (HPRT1), and ribosomal protein large P0 (RPLP0), the expression of which remained constant.

### 4.9. Statistical Analysis

All experiments have been carried out in triplicates. Mean and standard deviation were calculated. Statistical significative differences (at least *p* < 0.05) were assessed comparing different treatments and/or between old and young leaf results applying Student’s *t*-test (mean comparison) or Fisher-Snedecor test (variance comparison) using PAST3 software [72].

## 5. Conclusions

Our study lays the foundation for a potential biotechnological application of the invasive seagrass *H*. *stipulacea* that might fill the role of natural provider of antioxidant agent acting against oxidative stress in fibroblast cell lines. In agreement with other previous reports [10,11,17], this species might represent a novel valuable source for human health benefits. This might be a great opportunity since this species is a threat for coastal marine ecosystems because of its invasiveness. A careful harvesting of *H*. *stipulacea* from nature could bring up a synergy between the commercial exploitation for human wellbeing of the invader in areas where the species is not native and benefits the conservation of the natural capital and ecosystem health.

## Figures and Tables

**Table 1:** Protein, carbohydrate, and lipid concentrations (mg·g^−1^ dry leaf) of young and old leaves of *Halophila stipulacea.* Values are reported as mean ± S.D.

	Proteins	Carbohydrates	Lipids
Young leaves	21.3 ± 0.5	19.0 ± 0.6	5.8 ± 0.1
Old leaves	8.2 ± 0.6	10.9 ± 1.1	1.6 ± 0.3

## Data Availability

The data presented in this study are available on request from the corresponding author.

## Supplementary Materials

The following are available online at https://www.mdpi.com/1660-3397/19/1/37/s1. Table S1: List of the 84 oxidative stress-relied genes of the WI-38 cell line analyzed in our study (from: RT² Profiler™ PCR Array Human Oxidative Stress Plus (Product no. 330231; Cat. No. PAHS-065Y)). Table S2: Oxidative stress-relied genes of the WI-38 cell line upregulated (fold regulation > 2) or downregulated (fold regulation < −2) during treatments with old leaf extract or H_2_O_2_.
