# Supplementary material for: In Vitro Evaluation of Antioxidant Potential of the Invasive Seagrass Halophila stipulacea
Source: Mar Drugs. 2021 Jan 16;19(1):37. doi: 10.3390/md19010037 (PMC7830009; doi:10.3390/md19010037)
Supplement: Supplementary file 1 [file marinedrugs-19-00037-s001.pdf]

marine drugs

Supplementary material

In Vitro Evaluation of Antioxidant Potential of the Invasive Seagrass *Halophila stipulacea*

Clementina Sansone [1,*], Christian Galasso [1], Marco Lo Martire [2], Tomás Vega Fernández [1], Luigi Musco [1], Antonio Dell'Anno [2], Antonino Bruno [3], Douglas M. Noonan [4,5], Adriana Albini [5] and Christophe Brunet [1]

[1] Stazione Zoologica Anton Dohrn, Istituto Nazionale di Biologia, Ecologia e Biotecnologie Marine, Villa Comunale, 80121 Napoli, Italy; christian.galasso@szn.it (C.G.); tomas.vegafernandez@szn.it (T.V.F.); luigi.musco@szn.it (L.M.); christophe.brunet@szn.it (C.B.)
[2] Department of Life and Environmental Sciences, Università Politecnica delle Marche, Via Brecce Bianche, 60131 Ancona, Italy; m.lomartire@univpm.it (M.L.M.); a.dellanno@staff.univpm.it (A.D.)
[3] Unit of Immunology, IRCCS MultiMedica, 20138 Milan, Italy; antonino.bruno@multimedica.it (A.B.);
[4] Department of Biotechnology and Life Sciences, University of Insubria, 211000 Varese, Italy; douglas.noonan@uninsubria.it
[5] Laboratory of Vascular Biology and Angiogenesis, IRCCS MultiMedica, 20138 Milan, Italy; adriana.albini@multimedica.it (A.A.)
* Correspondence: clementina.sansone@szn.it; Tel: +39-0815833262

marine drugs

MDPI

Supplementary material

Table 1. List of the 84 oxidative stress-relied genes of the WI-38 cell line analyzed in our study (from: RT^{2} Profiler™ PCR Array Human Oxidative Stress Plus (Product no. 330231; Cat. No. PAHS-065Y)).

GENE SUPERFAMILY	GENE SUBFAMILY	GENES ANALYSED
**Antioxidants**	**Glutathione Peroxidases**	GPX1. GPX2. GPX3. GPX4. GPX5. GSTP1. GSTZ1.
	**Peroxiredoxins**	PRDX1. PRDX2. PRDX3. PRDX4. PRDX5. PRDX6 (AOP2).
	**Other Peroxidases**	CAT. CYBB. CYGB. DUOX1. DUOX2. EPX. LPO. MPO. PTGS1 (COX1). PTGS2 (COX2). TPO. TTN.
	**Other Antioxidants**	ALB. APOE. GSR. MT3. VIMP. SOD1. SOD3. SRXN1. TXNRD1. TXNRD2.
**Reactive Oxygen Species Metabolism**	**Superoxide Dismutases**	SOD1. SOD2. SOD3.
	**Other Superoxide Metabolism Genes**	ALOX12. CCS. DUOX1. DUOX2. MT3. NCF1. NCF2. NOS2 (INOS). NOX4. NOX5. UCP2.
	**Other Reactive Oxygen Species Metabolism Genes**	AOX1. BNIP3. EPHX2. MPV17. SFTPD.
	**Oxidative Stress Responsive Genes**	APOE. ATOX1. CAT. CCL5 (RANTES). CYGB. DHCR24. DUOX1. DUOX2. DUSP1 (PTPN16). EPX. FOXM1. FTH1. GCLC. GCLM. GPX1. GPX2. GPX3. GPX4. GPX5. GSR. GSS. HMOX1. HSPA1A (HSP70-1A). KRT1. LPO. MBL2. MPO. MSRA. NQO1. NUDT1. PDLIM1. PRDX2. PRDX5. PRDX6 (AOP2). PRNP. RNF7. VIMP. SEPP1. SIRT2. SOD1. SOD2. SQSTM1. SRXN1. TPO. TTN. TXN. TXNRD1. TXNRD2.
**Oxygen Transporters**		CYGB. MB.
**Pathway Activity Signature Genes**		AKR1C2. BAG2. FHL2. GCLM. GLA. HMOX1. HSP90AA1. LHPP. NCOA7. NQO1. PTGR1. SLC7A11. SPINK1. TRAPPC6A. TXN. TXNRD1.

marine drugs

MDPI

Supplementary material

Table S2: Oxidative stress-relied genes of the WI-38 cell line upregulated (fold regulation > 2; in red) or downregulated (fold regulation < - 2; in blue) during treatments with H_2O_2. old leaf extract alone. before and after hydrogen peroxide injury.

GENE	Description	H_2O_2 (10 mM)	Extract (10 µgml^{-1})	Extract added before H_2O_2	Extract added after H_2O_2
ALB	Albumin	15.4	13.2	43.4	27.5
APOE	Apolipoprotein E	4.2	3.6	2.5	-3.6
CCL5	Chemokine (C-C motif) ligand 5	1.6	1.1	62.5	9.7
CYBB	Cytochrome b-245	-38.0	3.9	-26.2	-18.5
DUOX1	Dual oxidase 1	-2.1	-1.1	-1.7	-2.4
DUOX2	Dual oxidase 2	-2.5	-1.1	-3.5	-4.6
EPHX2	Epoxide hydrolase 2. cytoplasmic	3.1	-19.3	-9.3	-6.6
EPX	Eosinophil peroxidase	2.0	-2.1	1.5	1.1
GCLC	Glutamate-cysteine ligase	2.1	-2.1	1.5	1.1
GPX2	Glutathione peroxidase 2	1.5	1.5	-1.3	-1.8
GPX5	Glutathione peroxidase 5	-1.7	1.1	-1.3	-1.6
GSR	Glutathione reductase	-5.7	1.1	2.3	3.2
HSPA1A	Heat shock 70kDa protein 1A	-1.9	-3.8	1.8	-1.2
KRT1	Keratin 1	1.2	1.1	-1.1	1.3
LPO	Lactoperoxidase	1.6	1.1	2.3	3.2
MB	Myoglobin	1.6	1.1	2.3	17.0
MBL2	Mannose-binding lectin 2	1.6	1.1	2.3	3.2
MPO	Myeloperoxidase	14.3	51.2	2.3	3.2
MT3	Metallothionein 3	8.7	37.0	15.4	2.0
NOS2	Nitric oxide synthase 2	1.6	1.1	2.3	3.2
NOX5	NADPH oxidase domain 5	-1.1	-1.4	-12.7	1.6
PRDX3	Peroxiredoxin 5	1.6	1.1	2.3	3.2
PTGS2	Prostaglandin-endoperoxide synthase 2	-1.3	1.7	-1.2	-1.5
SEPP1	Selenoprotein P. plasma. 1	-5.0	-1.1	-1.4	-3.3
SFTPD	Surfactant protein D	-1.4	2.8	-3.1	-1.5
SOD3	Superoxide dismutase 3	-62.8	-89.8	4.7	-30.6

marine drugs

MDPI

Supplementary material

TPO	Thyroid peroxidase	2.4	1.1	1.5	1.6
AKR1C2	Aldo-keto reductase member C2	1.6	1.1	2.3	16.7
SLC7A11	Solute carrier family 7 member 11	1.3	-1.2	-2.0	1.1
SPINK1	Serine peptidase inhibitor. Kazal type 1	1.4	-1.0	-1.4	1.0
TXNRD1	Thioredoxin reductase 1	1.6	1.1	2.3	3.2
